# Respiratory level tracking with visual biofeedback for consistent breath-hold level with potential application in image-guided interventions

**DOI:** 10.1186/s41747-018-0052-7

**Published:** 2018-09-05

**Authors:** W. J. Heerink, M. D. Dorrius, H. J. M. Groen, P. M. A. Van Ooijen, R. Vliegenthart, M. Oudkerk

**Affiliations:** 1 Center for Medical Imaging – North East Netherlands, University of Groningen, Groningen, The Netherlands; 2 Department of Radiology, University of Groningen, University Medical Center Groningen, Groningen, The Netherlands; 3 Department of Pulmonary Diseases, University of Groningen, University Medical Center Groningen, Groningen, The Netherlands

**Keywords:** Breath holding, Image-guided biopsy, Lung, Reproducibilty of results, Respiration

## Abstract

**Background:**

To present and evaluate a new respiratory level biofeedback system that aids the patient to return to a consistent breath-hold level with potential application in image-guided interventions.

**Methods:**

The study was approved by the local ethics committee and written informed consent was waived. Respiratory motion was recorded in eight healthy volunteers in the supine and prone positions, using a depth camera that measures the mean distance to thorax, abdomen and back. Volunteers were provided with real-time visual biofeedback on a screen, as a ball moving up and down with respiratory motion. For validation purposes, a conversion factor from mean distance (in mm) to relative lung volume (in mL) was determined using spirometry. Subsequently, without spirometry, volunteers were given breathing instructions and were asked to return to their initial breath-hold level at expiration ten times, in both positions, with and without visual biofeedback. For both positions, the median and interquartile range (IQR) of the absolute error in lung volume from initial breath-hold were determined with and without biofeedback and compared using Wilcoxon signed rank tests.

**Results:**

Without visual biofeedback, the median difference from initial breath-hold was 124.6 mL (IQR 55.7–259.7 mL) for the supine position and 156.3 mL (IQR 90.9–334.7 mL) for the prone position. With the biofeedback, the difference was significantly decreased to 32.7 mL (IQR 12.8–59.6 mL) (*p* < 0.001) and 22.3 mL (IQR 7.7–47.0 mL) (*p* < 0.001), respectively.

**Conclusions:**

The use of a depth camera to provide visual biofeedback increased the reproducibility of breath-hold expiration level in healthy volunteers, with a potential to eliminate targeting errors caused by respiratory movement during lung image-guided procedures.

## Key points

- A depth camera can be used to accurately monitor the level of respiration
- Visual feedback enables volunteers to hold their breath at a consistent level
- This method can have potential application in lung image-guided interventional procedures, reducing targeting errors caused by respiration

## Background

Tumour movement caused by patient respiration can be a serious problem during image-guided interventional procedures. Lung nodules near the diaphragm, for example, typically move > 2 cm with inspiratory capacity [1]. When tissue diagnosis of a suspicious lung nodule by computed tomography (CT)-guided biopsy is required, a predictable nodule position is important. Often, giving a patient specific breathing instructions is not sufficient to facilitate a consistent breath-hold level. When patients have difficulty returning to their initial breath-hold level, accurate targeting of smaller nodules becomes impossible.

Currently available respiratory tracking systems suitable for image-guided intervention consist of respiratory belts that are cumbersome to install, only have a weak correlation with nodule position and do not adjust for a change in breathing pattern [2]. Several groups have investigated the use of a depth camera to monitor patient respiratory motion for four-dimensional radiotherapy planning [3–6]. Depth cameras measure the distance to a surface for each pixel and can thus be used to determine changes in skin surface, caused by respiratory motion, in real time [7].

In this study, we implemented and tested a similar setup, in combination with real-time visual biofeedback to the patient. The aim was to present and evaluate a new respiratory level biofeedback system that aids patients to return to a consistent level of breath-hold with potential application in image-guided interventions.

## Methods

### The setup

**Fig. 1:**
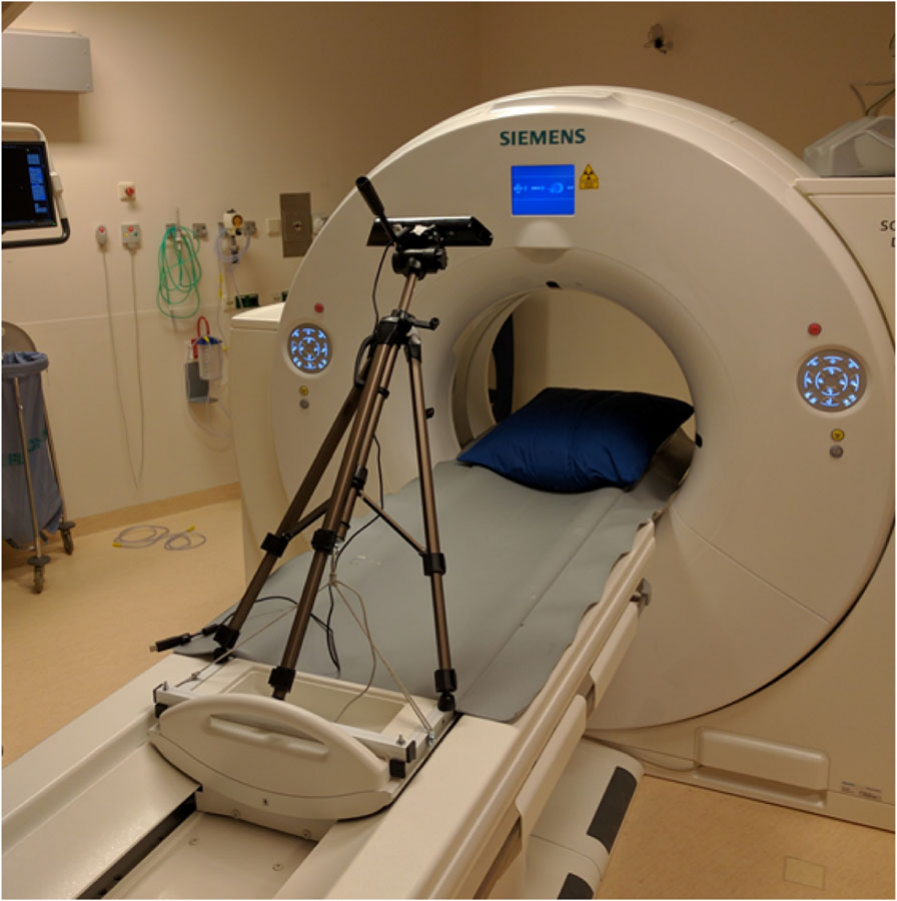
CT table setup. Setup of the Kinect camera on a CT table, positioned with its field of view into the gantry. The tablet can provide visual biofeedback to patient and operator

**Fig. 2:**
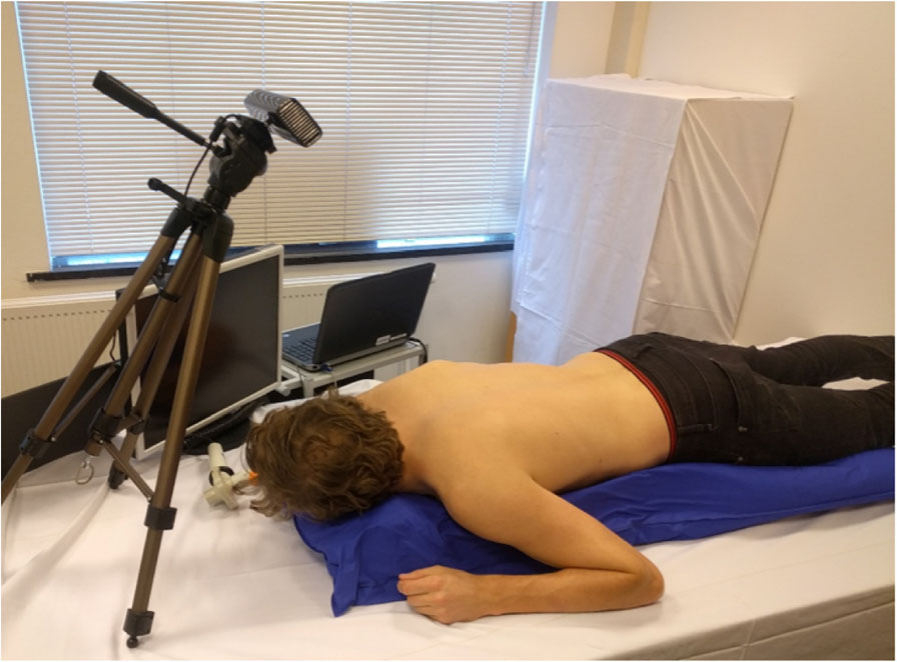
Experimental setup. Setup of how the Kinect was used in this study. Volunteers could see the visual biofeedback on the additional screen

The Kinect for Windows V1 (Microsoft, Redmond, VA, USA) was used to measure respiratory surface movement. It can be positioned onto the CT table using a setup with an adapted tripod as shown in Fig. 1, where it remains stationary relative to the patient. The Kinect was angled forward towards the patient, so the patient remained in its field of view, while in the CT gantry. For convenience and lack of continuous access to a CT scanner, all measurements were performed on a regular table, with a setup simulating that of a CT table with CT gantry (Fig. 2).

### Software

**Fig. 3:**
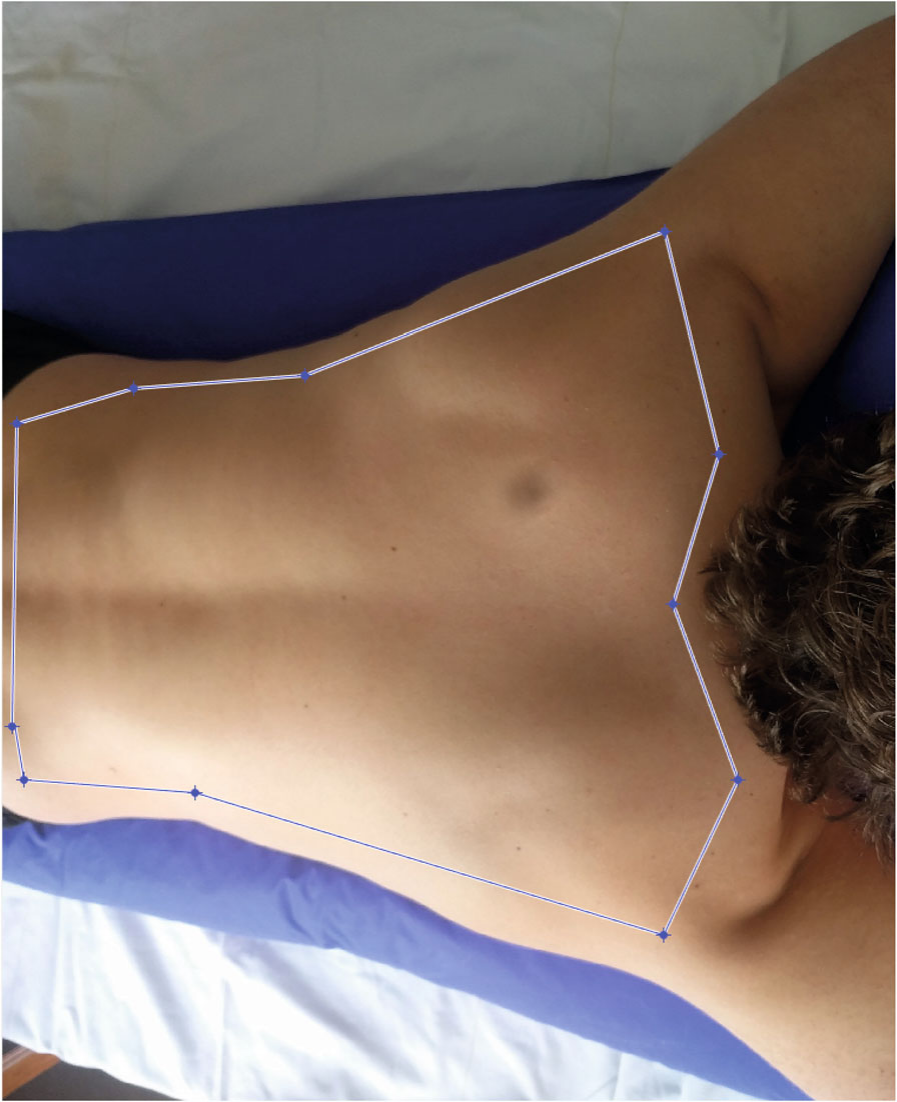
Screenshot of ROI selection process. The *colour image* is captured by the Kinect and the *blue lines* represent the border of the selected ROI

**Fig. 4:**
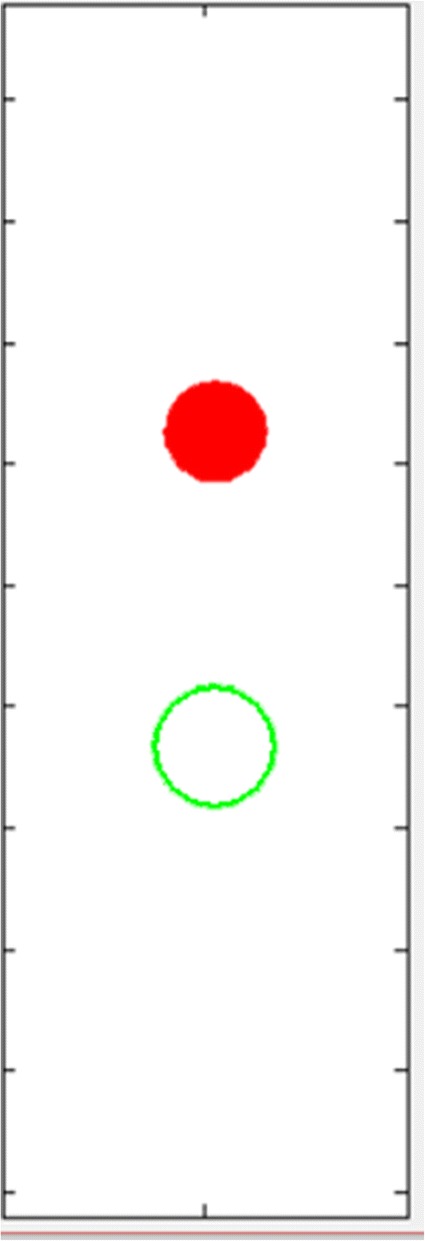
Screenshot of biofeedback provided to the volunteers. The *red circle* moves up and down with respiration and the *green circle* corresponds to the respiratory level, previously set

The Kinect provides a depth map and a colour image of the scene. Using Matlab 2014a’s Image Acquisition Toolkit (the Mathworks, Natick, MA, USA) and an in-house written script, these feeds were processed. The operator interacted with this script using a graphical user interface. First, the colour image was used to interactively select a polygonal region of interest (ROI) outlining the abdomen and thorax, including as much as possible from the visible skin surface. This step takes approximately 5 s. Figure 3 shows a screenshot of the ROI selection process. Erroneous data pixels (identified as distance = 0) were excluded and the mean distance to the entire ROI was calculated. The mean distance and corresponding time point were saved for every frame, with a frame rate of approximately 15 Hz. After the selection of the ROI, the preferred level of expiration was set using the graphical user interface. When this was selected, the volunteers received visual biofeedback of the level of expiration: on a screen next to the volunteer, a red circle moved up and down with respiration and a green circle corresponded to the respiratory level, previously set (Fig. 4).

### Volunteer experiments

The study has been reviewed and the need for written informed consent was waived by the Medical Ethics Review Board of University Medical Center Groningen (number 2017/226). Initial validation of the accuracy of the depth measurements as a measure of respiratory level was performed on a single healthy volunteer. He was positioned on a vacuum mattress (BodyFIX BlueBAG, Elekta, Crawley, UK) to limit movement and maximise the respiratory surface motion on the skin surface and asked to perform several respiratory manoeuvres, in the supine and prone positions. During these manoeuvres, the mean depth measurements were recorded and a Jaeger Masterscope with spirometry software (SentrySuite V2.13, CareFusion, San Diego, CA, USA) was used to measure the respiratory level. The spirometer was calibrated using a 3 L calibration syringe. Local ambient temperature, humidity and air pressure were updated in the system. The respiratory manoeuvres consisted of a complete inspiration, resulting in a peak in both signals to temporally align them, and several tidal volumes. The volume and inverted mean distance measurements for the supine and prone positions were superimposed plotted in graphs and assessed visually.

To evaluate whether the visual biofeedback can facilitate the return to a predictable and consistent level of breath-hold, the system was tested with eight healthy volunteers (four men, four women). All volunteers had their height and weight measured and their body mass index was calculated. After the volunteers were positioned correctly on the table, the air was vacuumed out of the mattress while making sure the volunteers’ sides were fully supported by the mattress, too.

Volunteers were randomly positioned in the supine or prone position first. Next, a conversion factor from mm to mL was determined before receiving biofeedback. This was performed by measuring the change in lung volume simultaneously with the change in mean distance to the ROI. The minimal and maximal values of both signals of four corresponding tidal volumes around functional residual capacity (FRC) level were extrapolated from the graphs and averaged. The mean tidal volume (in mL) was subsequently divided by the mean tidal movement (in mm) to determine a conversion factor C (mL/mm) for every volunteer for both positions.

Subsequently, without spirometry, the volunteers were given breath-hold instructions: ‘Breathe out…, breathe in…, breathe out and hold your breath’. This way, the volunteers held their breath at FRC, approximately. In fact, breath-hold at a lower lung volume results in less organ motion compared to inspiration, because of a decreased gas exchange in the alveoli [8–10]. Additionally, the British Thoracic Society advises breath-hold at FRC level (gentle expiration) for biopsy of nodules in the lung base [11].

Then, the volunteers were asked ten times to return to the same level of breath-hold for approximately 5 s, with 30s intervals. This was performed twice, in a random order: once while the volunteers received the same breathing instruction but could not see the visual biofeedback; and once while they could see the screen with biofeedback. They were asked to slowly breathe out, to prevent any overshoot, until the red ball was inside the green circle and then hold their breath. Additionally, they were instructed not to correct their breath-hold in case they did overshoot the target level.

### Variables and statistical analysis

Median conversion factors were determined for volunteer position (C_supine_ and C_prone_) and sex (C_males_ and C_females_). The periods of breath-hold were selected and their mean values determined using a graphical data selection tool in Matlab. The absolute error (E) between the initial mean level of breath-hold and the consequent attempts to return to said level were saved. These values were converted from distance to volume using the corresponding conversion factor.

Normality of data distribution was evaluated with the Shapiro-Wilk test. Due to non-normal distribution, median and interquartile range (IQR) of the absolute error were determined for the measurements with and without feedback overall, by sex, by position (supine and prone) and by volunteer. Paired variables (with feedback vs without feedback) were compared using Wilcoxon signed rank tests, paired by the volunteer’s breath-hold attempt and unpaired variables (men vs women, supine vs prone) were compared using Mann-Whitney *U* tests. Box plots were made for the results in the supine and prone positions, grouped per volunteer, comparing with and without feedback.

All measurements were performed in Matlab and results were subsequently imported in SPSS 23.0 (IBM, New York, NY, USA) to perform statistical analysis. Level of significance was set at *p* < 0.050.

## Results

**Fig. 5:**
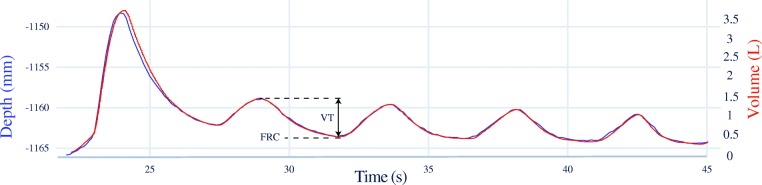
Supine position. Superimposed graphs of mean depth, measured by the Kinect (*blue*, left axis) and volume, measured with spirometry (*red*, right axis) of a volunteer in the supine position. Periods of breath-hold are indicated with *grey boxes*. *FRC* functional residual capacity, *VT* tidal volume

**Fig. 6:**
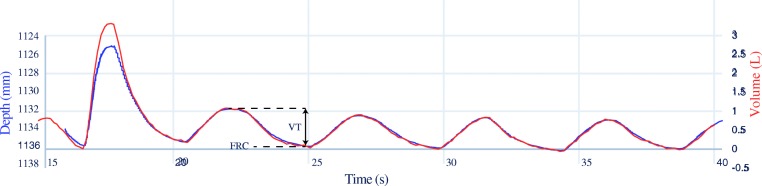
Prone position. Superimposed graphs of mean depth, measured by the Kinect (*blue*, left axis) and volume, measured with spirometry (*red*, right axis) of a volunteer in prone position. Periods of breath-hold are indicated with *grey boxes*. *FRC* functional residual capacity, *VT* tidal volume

The median age of the volunteers was 29 years (IQR 25–33 years), with a median body mass index of 21.5 kg/m^2^ (IQR 20.9–23.3 kg/m^2^). Figures 5 and 6 show the graphs of the initial validation measurements of a single volunteer in the supine and prone positions, respectively. The measurements performed with the Kinect system and with the spirometer are superimposed on each other.

All parameters were non-normally distributed (*p* < 0.001). The conversion factors did not differ between volunteer positions (C_supine_ = 187.8 mL/mm, C_prone_ = 164.7 mL/mm; *p* = 0.767) or between men and women (C_males_ = 174.1 mL/mm, C_females_ = 158.9 mL/mm; *p* = 0.452).

**Fig. 7:**
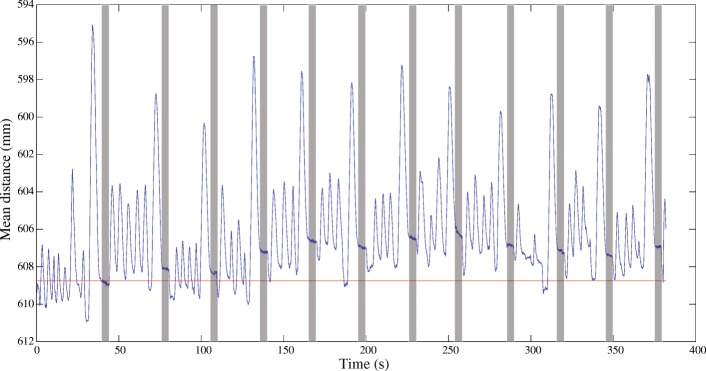
Respiratory level without feedback. Respiratory level of a volunteer (F4) in the prone position, without visual feedback. *Red line* represents the initial level of breath-hold. The volunteer was given breathing instruction every 30 s and asked to return to the same respiratory level and maintain breath-hold for a couple of s, indicated by the short horizontal periods

**Fig. 8:**
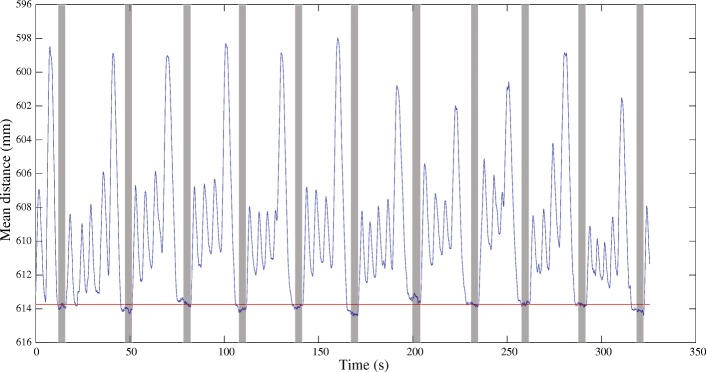
Respiratory level with feedback. Respiratory level of a volunteer (F4) in the prone position, with visual feedback. *Red line* is the initial level of breath-hold. Volunteer was asked to return to the same respiratory level every 30 s with the aid of visual biofeedback and maintain breath-hold for a couple of s, indicated by the short horizontal periods

**Fig. 9:**
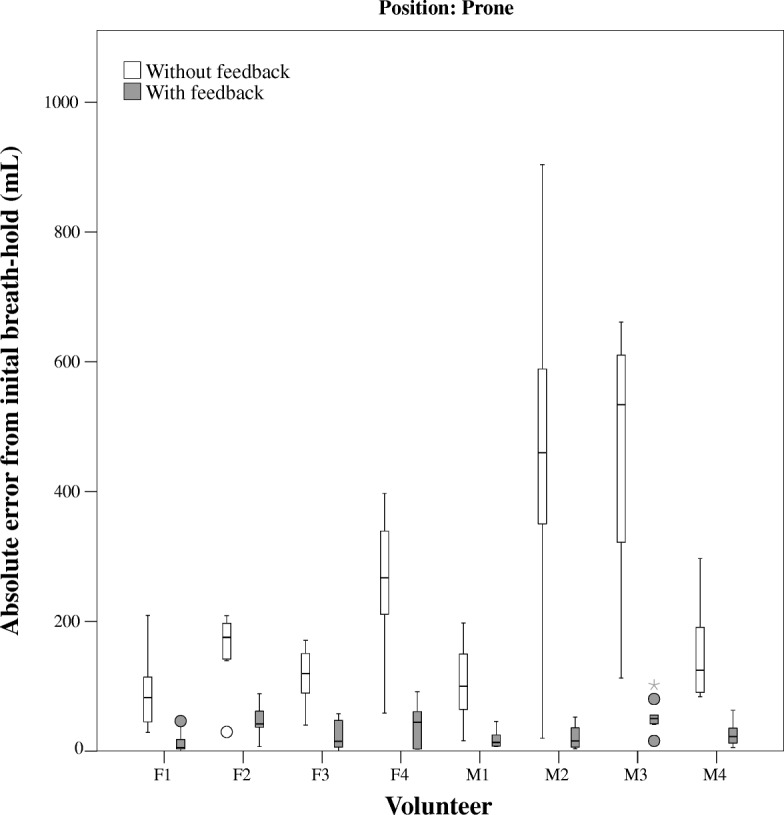
Breath-hold error in prone position. Box plots of the error from initial breath-hold, without and with biofeedback, grouped by volunteer, in the prone position

**Fig. 10:**
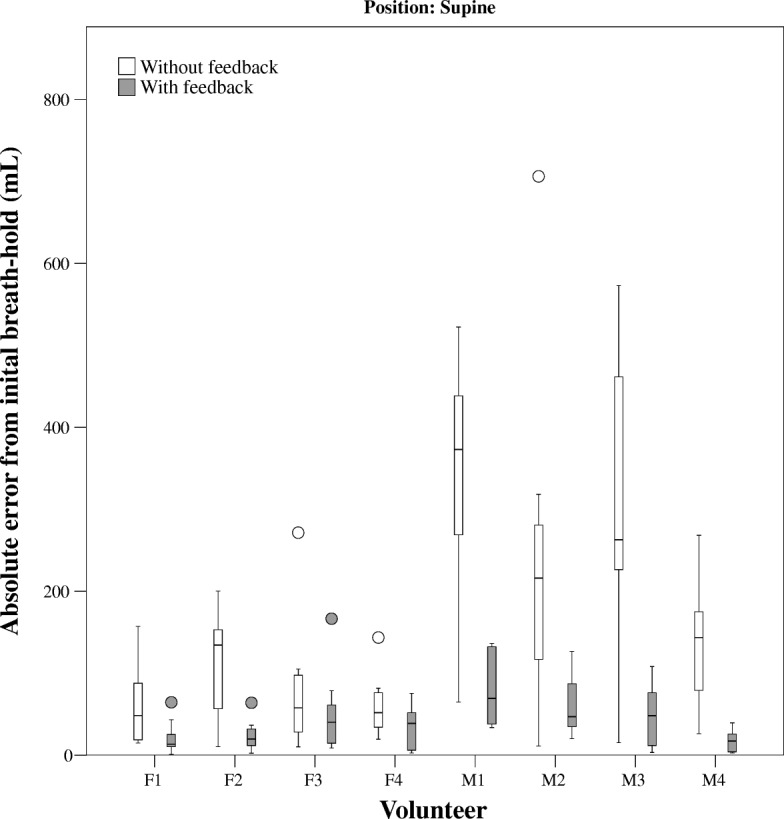
Breath-hold in supine position. Box plots of the error from initial breath-hold, without and with biofeedback, grouped by volunteer, in the supine position

Figures 7 and 8 show graphs of the respiratory level of a volunteer (F4) in the prone position, without and with biofeedback, respectively. Figures 9 and 10 show box plots of the all the individual volunteers, without and with biofeedback, grouped by volunteer, for the supine and prone positions, respectively.

For all volunteers, the absolute error from initial breath-hold at FRC level reduced from 147.6 mL (IQR 76.8–276.8 mL) without feedback to 27.7 mL (IQR 10.9–51.7 mL) with feedback (*p* < 0.001). Table 1 shows the absolute error for both sexes, in both positions, stratified by feedback. The error without feedback was higher in the prone position (*E*_*prone*_ = 156 mL vs *E*_*supine*_ = 125 mL, *p* = 0.012). With feedback, there was no significant difference between the two volunteer positions, though the error trended to be lower in the prone position (*E*_*prone*_ = 22 mL vs *E*_*supine*_ = 32 mL, *p* = 0.086).

**Table 1:** Absolute error from initial breath-hold at expiration

	Median	Q1–Q3	*p* value
Skin surface error without feedback (mm)	0.79	0.47–1.56	< 0.001
Skin surface error feedback (mm)	0.15	0.06–0.30	
*E*_*overall*_ without feedback (mL)	147	76.8–276.8	< 0.001
*E*_*overall*_ with feedback (mL)	27.7	10.9–51.7	
*E*_*men*_ with feedback (mL)	35.5	14.3–52.3	0.081
*E*_*women*_ with feedback (mL)	23.7	8.2–50.4	
*E*_*supine*_ without feedback (mL)	124.6	55.7–259.7	0.012
*E*_*prone*_ without feedback (mL)	156.3	90.9–334.7	
*E*_*supine*_ with feedback (mL)	31.7	12.8–59.6	0.086
*E*_*prone*_ with feedback (mL)	22.3	7.7–47.0	

*E* absolute error from initial breath-hold at functional residual capacity level

## Discussion

The aim of this study was to present and evaluate a new respiratory level biofeedback system that aids patients to return to a consistent level of breath-hold with potential application to image-guided interventions. We demonstrated that the system described in this paper enables healthy volunteers to return to 28 mL of their initial breath-hold, which is a significant reduction from the 147 mL that they managed without the biofeedback system.

Without feedback, the volunteers had a larger absolute error in the prone position compared to the supine position (*E*_*prone*_ = 156 mL vs *E*_*supine*_ = 125 mL, *p* = 0.012). This illustrates that it is harder for patients to get to the same level of breath-hold while lying on their stomach. With feedback, the volunteers no longer had increased difficulty with the prone position compared to the supine position. In fact, they seemed to perform better in the prone position (*E*_*prone*_ = 22 mL vs *E*_*supine*_ = 32 mL, *p* = 0.086). We speculate this is because the back provides a more stable platform to measure a mean distance to because there is less soft tissue such as fat and breast tissue to impair the measurements. In our department, approximately half of the CT-guided lung biopsies are performed in the prone position, so for these procedures visual biofeedback will be of increased importance.

When targeting a lesion in image-guided interventions, it is not really a reliable, consistent lung volume that is important. For a radiologist, it is about the target being in the same position to when the image was acquired. However, to analyse whether the system presented here results in a reproducible target position would require using a CT scanner, resulting in a radiation dose in healthy volunteers. Therefore, this was not an option for this study. Using CT, Chen et al. [1] investigated the motion of lung nodules from full inspiration to end-expiration during tidal volume breathing (i.e. inspiratory capacity). The average motion of all 85 included nodules was 17.6 mm; in the left and right lower lobes, this was 23.8 mm and 25.3 mm, respectively. The average inspiratory capacity of men and women was 3.5 L and 2.4 L, so considering a linear relation, a lung volume change of 100 mL would result in a nodule motion of 1.1 mm and 0.7 mm in the lower lobes, for men and women, respectively [12]. Translating these numbers to the results of this study, one can conclude that even in the lower lung lobes, the biofeedback system can potentially enable men and women to have a predictable consistent nodule position of well below 0.5 mm. Even when considering nodules with extreme respiratory motion from the study by Chen et al. [1] (up to 60 mm), this would result in a reproducibility of the nodule position of within 1 mm.

Price et al. [6] have recently performed a clinical trial to assess the feasibility of using an in-house developed optical surface tracking device to facilitate consistent breath-hold during radiation therapy. They found that patients were able to tolerate the feedback well and that they had a moderately improved reproducibility of skin surface. They used traffic light colours to provide visual feedback to the patients and were able to reduce the mean amplitude of skin movement from 2.0 mm to 1.7 mm. In a previous healthy volunteer study [13], they achieved an improvement from 1.4 to 0.6 mm. In our study, the skin movement of the volunteers improved from 0.79 to 0.15 mm, when providing the feedback. Though these measurements cannot be directly compared, because they rely on technical factors as camera angle, our system should have a relatively higher rate of improvement.

The Kinect has the added benefit of being a generally available, low-cost system. Several groups have analysed the feasibility of using the Kinect camera to monitor respiratory motion for respiratory gated or four-dimensional CT-based continuous radiotherapy [3–5]. Though the results seem promising, to our knowledge, no clinical studies utilising the Kinect have been published yet. The Kinect-based respiratory motion monitoring systems are mostly compared with the RPM Gating System (Varian Medical System, Palo Alto, CA, USA), a clinically available respiratory motion tracking system that utilised the movement of a marker box placed on the patient’s chest to gate radiation therapy. This system is not suitable for interventional procedures because the box has to be placed on disinfected skin and can easily be knocked out of place. As it only tracks the movement of a single marker, it would not be able to detect a change in breathing pattern either. During interventional procedures, patients are more likely to alter from thoracic to abdominal breathing, or vice versa, rendering the tracking inaccurate. The value of skin surface motion tracking in combination with a tightly positioned vacuum mattress is that all respiratory movement can be visualised and thus be used as patient feedback.

A change in breathing pattern is also a problem when using abdominal/chest belts. These belts measure the circumference of the patient’s chest or abdomen to provide patient feedback. Schoth et al. [14, 15] reported reduced intervention time and radiation exposure using the IBC system (Mayo Clinic Medical Devices, USA) for CT-guided lung biopsy while Carlson et al. [14, 15] reported a reduction in targeting attempts using this belt in CT fluoroscopy-guided lung biopsy. However, in our experience these belts are cumbersome to setup and unreliable. In a review of another bellows belt system (Philips Medical Systems, Eindhoven, The Netherlands), Locklin et al. [2] demonstrated only a weak correlation between chest circumference and nodule position.

Another option that has been considered is the use of spirometry to monitor lung volume. Tomiyama et al. [16] used a respiratory monitor to trigger an electric light bulb as an indication of a similar level of breath-hold, resulting in a high diagnostic accuracy (96%) in CT-guided biopsy of small (< 15 mm) lung nodules. The active breathing coordinator system (Elekta Instrument AB, Stockholm, Sweden) is a clinically available system used to actively monitor lung volume and suspend the patient’s breathing. A valve closes and holds respiration at a certain level, to facilitate consisted tumour position for gated radiotherapy. From a practical standpoint, spirometry seems less suited for interventional procedures, because it prevents communication from patient to physician. If any breath were to escape from the mouthpiece, the procedure would no longer be reliable.

As an alternative to CT-guided lung biopsy, CT fluoroscopy can be used to target lung lesions. The advantage of CT fluoroscopy is the real-time feedback it provides so the radiologist can verify the lesions’ position and immediately place the biopsy needle. It has been reported with similar diagnostic accuracy and lower complication rates, compared to CT-guided lung biopsy [17]. However, both patient and radiologist experience a higher exposure to radiation, which is a significant disadvantage. Additionally, depending on the CT scanner’s gantry tilt, the biopsy needle path is limited to in-plane approaches.

During the biofeedback evaluation, the volunteers were not breathing into a spirometer. Instead, the spirometer was used during validation measurements beforehand, to determine a conversion factor from chest height to lung volume. When we attempted to use the spirometer during the feedback evaluation, some volunteers demonstrated difficulty in maintaining breath-hold because the mount piece prevented them from closing their mouth, as if they were not able to close their glottis. Moreover, the spirometer data showed a significant drift in the long-term measurements, even after rigorous (re)calibration of the spirometer, rendering these long-term spirometry data unusable. Of note, the spirometry measurements are not required for the system to function in clinical practice. These were performed only for validation of the system.

There are some limitations to this study. With a mean age of 29 years and a mean body mass index of 22.2, the volunteers were all young healthy adults, compared to the potential target group. The breathing instructions resulted in a breath-hold at FRC level. Obese patients generally breathe at a lower FRC [18] and patients suffering from chronic obstructive pulmonary disease breathe at a higher FRC than healthy volunteers [19]. Although the relation between chest wall motion and diaphragmatic excursion is approximately linear in healthy adults, this might not be true for patients [20]. Additionally, Harte et al. [21] have shown that patients with cystic fibrosis have a lower correlation between chest wall movement and lung volume changes. Although differences in tracking accuracy are to be expected between the volunteers and patients, the procedure with biofeedback is feasible with low error rates and easy to instruct to volunteers.

Patients might arguably have more difficulty in interpreting the biofeedback and therefore in returning to their initial level of breath-hold every time. This does not have to lead to targeting errors per se, because the operator will also see the biofeedback. He/she can therefore keep on instructing the patient until the patient manages to hold his breath is at the level the CT scan was acquired, before proceeding with needle manipulation. It should also be considered that patients who are difficult to instruct will have more difficulty to return to a consistent level of breath-hold with only breathing instructions, so these patients might benefit even more from the feedback system.

Often, in interventional procedures parts of the thorax and abdomen have to be covered with sterile drapes. Movement of these drapes would result in an error in the depth measurements. This can be prevented by either using a drape with a large hole, and disinfecting a larger surface of the skin, or by using surgical incise drape with adhesive backing. This facilitates a larger ROI to be selected, without having to include loose fitting drapes.

In conclusion, we presented a method to provide patients with visual biofeedback of their respiratory level, to enable them to return to a consistent level of breath-hold during image-guided interventions. The depth measurements have proven to be an accurate measure of lung volume and the visual biofeedback enabled healthy volunteers to return to 28 mL of their initial breath-hold at expiratory level, corresponding to an estimated target position reproducibility of < 0.5 mm. If implemented in an image-guided intervention suite, it has the potential to prevent targeting errors caused by respiratory motion and thereby to increase targeting accuracy.

## Abbreviations

C: Conversion factor
CT: Computed tomography
D: Difference
E: Absolute error
FRC: Functional residual capacity
IQR: Interquartile range
ROI: Region of interest

## References

[1] Chen A, Pastis N, Furukawa B, Silvestri GA (2015) The effect of respiratory motion on pulmonary nodule location during electromagnetic navigation bronchoscopy. Chest 147:1275–1281 10.1378/chest.14-1425 25357229

[2] Locklin JK, Yanof J, Luk A, Varro Z, Patriciu A, Wood BJ (2007) Respiratory biofeedback during CT-guided procedures. J Vasc Interv Radiol 18:749–755 10.1016/j.jvir.2007.03.010 PMC2562717 17538137

[3] Tahavori F, Alnowami M, Wells K (2014) Marker-less respiratory motion modeling using the Microsoft Kinect for windows. SPIE Medical Imaging 1–10

[4] Lim SH, Golkar E, Rahni AAA (2014) Respiratory motion tracking using the Kinect camera. IEEE Conference on Biomedical Engineering and Sciences (IECBES). 10.1109/IECBES.2014.7047619

[5] Ortmüller J, Gauer T, Wilms M, Handels H, Werner R (2015) Respiratory surface motion measurement by Microsoft Kinect. Current Directions in Biomedical Engineering 1:270–273

[6] Price GJ, Faivre-Finn C, Stratford J et al (2017) Results from a clinical trial evaluating the efficacy of real-time body surface visual feedback in reducing patient motion during lung cancer radiotherapy. Acta Oncol 57:211–218 10.1080/0284186X.2017.1360511 28780900

[7] Liu HH, Koch N, Starkschall G et al (2004) Evaluation of internal lung motion for respiratory-gated radiotherapy using MRI: part II—margin reduction of internal target volume. Int J Radiat Oncol Biol Phys. 60:1473–1483 10.1016/j.ijrobp.2004.05.054 15590178

[8] Holland AE, Goldfarb JW, Edelman RR (1998) Diaphragmatic and cardiac motion during suspended breathing: preliminary experience and implications for breath-hold MR imaging. Radiology 209:483–489 10.1148/radiology.209.2.9807578 9807578

[9] Lanphier EH, Rahn H (1963) Alveolar gas exchange during breath holding with air. J Appl Physiol 18:478–482 10.1152/jappl.1963.18.3.478 31088045

[10] Lens E, Gurney-Champion OJ, Tekelenburg DR et al (2016) Abdominal organ motion during inhalation and exhalation breath-holds: pancreatic motion at different lung volumes compared. Radiother On 10.1016/j.radonc.2016.09.012 27773445

[11] Manhire A, Charig M, Clelland C et al (2003) Guidelines for radiologically guided lung biopsy. Thorax 58:920–936 10.1136/thorax.58.11.920 PMC1746503 14586042

[12] Barret KE, Ganong WF (2012). Ganong’s review of medical physiology.

[13] Parkhurst JM, Price GJ, Sharrock PJ, Jackson ASN, Stratford J, Moore CJ (2013) Self-Management of Patient Body Position, pose, and motion using wide-field, real-time optical measurement feedback: results of a volunteer study. Int J Radiat Oncol Biol Phys 87:904–910 10.1016/j.ijrobp.2013.08.048 24125700

[14] Schoth F, Plumhans C, Kraemer N et al (2010) Evaluation of an interactive breath-hold control system in CT-guided lung biopsy. Rofo 182:507–511 10.1055/s-0029-1245141 20143287

[15] Carlson SK, Felmlee JP, Bender CE et al (2005) CT fluoroscopy–guided biopsy of the lung or upper abdomen with a breath-hold monitoring and feedback system: a prospective randomized controlled clinical trial. Radiology 237:701–708 10.1148/radiol.2372041323 16244278

[16] Tomiyama N, Mihara N, Maeda M et al (2000) CT-guided needle biopsy of small pulmonary nodules: value of respiratory gating. Radiology 217:907–910 10.1148/radiology.217.3.r00dc10907 11110962

[17] Kim GR, Hur J, Lee SM et al (2011) CT fluoroscopy-guided lung biopsy versus conventional CT-guided lung biopsy: a prospective controlled study to assess radiation doses and diagnostic performance. Eur Radiol 21:232–239 10.1007/s00330-010-1936-y 20730613

[18] Parameswaran K, Todd DC, Soth M (2006). Altered respiratory physiology in obesity. Can Respir J.

[19] Barnes PJ, Drazen JM, Rennard SI, Thomson NC (2009) Asthma and COPD basic mechanisms and clinical management. Elsevier Ltd, San Diego

[20] Wang H-K, Lu T-W, Liing R-J, Shih TT-F, Chen S-C, Lin K-H (2009) Relationship between Chest Wall motion and diaphragmatic excursion in healthy adults in supine position. J Formos Med Assoc 108:577–586 10.1016/S0929-6646(09)60376-4 19586832

[21] Harte JM, Golby CK, Acosta J et al (2016) Chest wall motion analysis in healthy volunteers and adults with cystic fibrosis using a novel Kinect-based motion tracking system. Med Biol Eng Comput 54:1631–1640 10.1007/s11517-015-1433-1 PMC5069336 26872677

